# Mutations in porin LamB contribute to ceftazidime-avibactam resistance in KPC-producing Klebsiella *pneumoniae*

**DOI:** 10.1080/22221751.2021.1984182

**Published:** 2021-11-02

**Authors:** Yingyi Guo, Ningjing Liu, Zhiwei Lin, Xiaoliang Ba, Chuyue Zhuo, Feifeng Li, Jiong Wang, Yitan Li, Likang Yao, Baomo Liu, Shunian Xiao, Ying Jiang, Chao Zhuo

**Affiliations:** a Guangzhou Institute of Respiratory Health, First Affiliated Hospital of Guangzhou Medical University, Guangzhou, People’s Republic of China; b Laboratory of Respiratory Disease, People's Hospital of Yangjiang, Guangdong, People’s Republic of China; c Department of Veterinary Medicine, University of Cambridge, Cambridge, UK

**Keywords:** Ceftazidime-avibactam, KPC, LamB, PBP3, expression level of *bla*_KPC_

## Abstract

Ceftazidime-avibactam (CAZ-AVI) shows promising activity against carbapenem-resistant *Klebsiella pneumoniae* (CRKP), however, CAZ-AVI resistance have emerged recently. Mutations in KPCs, porins OmpK35 and/or OmpK36, and PBPs are known to contribute to the resistance to CAZ-AVI in CRKP. To identify novel CAZ-AVI resistance mechanism, we generated 10 CAZ-AVI-resistant strains from 14 CAZ-AVI susceptible KPC-producing *K. pneumoniae* (KPC-Kp) strains through *in vitro* multipassage resistance selection using low concentrations of CAZ-AVI. Comparative genomic analysis for the original and derived mutants identified CAZ-AVI resistance-associated mutations in KPCs, PBP3 (encoded by *ftsI*), and LamB, an outer membrane maltoporin. CAZ-AVI susceptible KPC-Kp strains became resistant when complemented with mutated *bla*_KPC_ genes. Complementation experiments also showed that a plasmid borne copy of wild-type *lamB* or *ftsI* gene reduced the MIC value of CAZ-AVI in the induced resistant strains. In addition, *bla*_KPC_ expression level increased in four of the six CAZ-AVI-resistant strains without KPC mutations, indicating a probable association between increased *bla*_KPC_ expression and increased resistance in these strains. In conclusion, we here identified a novel mechanism of CAZ-AVI resistance associated with mutations in porin LamB in KPC-Kp.

## Introduction

Carbapenem-resistant *Klebsiella pneumoniae* (CRKP), an opportunistic pathogen spreading worldwide, is increasingly being isolated clinically and has caused numerous nosocomial outbreaks [1–4]. In 2020, 22% of *K. pneumoniae* strains isolated from patients in China were reported to be carbapenem-resistant [5]. In Greece, Italy, and the United States, the proportion of CRKP among *K. pneumoniae* is as high as 62%, 33%, and 11%, respectively [6,7]. CRKP is resistant to most of the commonly used antimicrobial agents, thus limiting treatment options and posing a serious threat to the global public health [8]. Carbapenemases are the main causes of multidrug resistance in CRKP and are classified into class A (KPC), class B (NDM, IMP, and VIM), and class D (OXA-48-like) [9]. In China and elsewhere, KPCs are major contributors to carbapenem resistance in *K. pneumoniae* and more than 70% of carbapenemase-producing *K. pneumoniae* produces KPCs [9–11].

Ceftazidime-avibactam (CAZ-AVI) is a cephalosporin/β-lactamase inhibitor combination that was approved by the US Food and Drug Administration (FDA) and the European Medicines Agency (EMA) in 2015 for the treatment of complicated urinary tract infections and complicated intra-abdominal infections in adults [12]. AVI is a novel non-β-lactam β-lactamase inhibitor that mainly inhibits class A and class C β-lactamases and has no activity against class B β-lactamases. Compared with other β-lactamase inhibitors, such as vaborbactam and relebactam, AVI has some unique advantages, including a long half-life, low molecular weight, strong polarity and the ability to interact with important catalytic residues near the active site of β-lactamase [13]. CAZ has a broad-spectrum antimicrobial activity and is capable of inhibiting the growth of bacteria by binding to the penicillin-binding proteins (PBP) of gram-negative bacteria and inhibiting cell wall synthesis [14–16]. Previous studies have shown that, compared with other β-lactam antibiotics, CAZ significantly and effectively expands its range of antibacterial activity when combined with AVI, especially against carbapenemase-producing bacteria, like *Enterobacteriaceae* and *P. aeruginosa* [17,18]. Previous studies conducted in multiple regions indicated that more than 80% of CRKP were sensitive to CAZ-AVI. A survey conducted in China in 2020 reported that all KPCs or OXA-48-producing CRKP strains were CAZ-AVI sensitive. Thus, CRKP, especially those producing KPCs or OXA-48, can be effectively treated by using CAZ-AVI [5,19–21].

However, resistance to CAZ-AVI in CRKP has been increasingly reported worldwide since 2016 [20,22–25]. Mutations in the Ω loop, an important active site of β-lactamases, are major contributors to CAZ-AVI resistance [20,22,23,26]. CRKP also develops resistance to CAZ-AVI through mutations in the porins OmpK35 and/or OmpK36 [23,27,28]. A report showed that the insertion of four amino acids in PBP3 (encoded by *ftsI*), the primary target of CAZ, increases the minimum inhibitory concentration (MIC) of CAZ-AVI [29]. Apart from these mentioned above, other unknown resistance mechanisms in clinical isolates need to be investigated. The main purpose of this study was to uncover the resistance mechanism of CAZ-AVI by inducing resistance in clinical strains of KPC-producing *K. pneumoniae* (KPC-Kp) through *in vitro* passaging under low antibiotic concentrations.

## Materials and methods

### Strains, antibiotics, and antimicrobial susceptibility testing

All test strains included in this study were isolated from patients admitted to the tertiary hospitals in Guangdong province during 2016–2019. All strains were identified as *K. pneumoniae* by a VITEK 2 Compact system (BioMérieux, Marcy l'Etoile, France). Polymerase chain reactions (PCRs) were conducted to identify the presence of *bla*_KPC_ [30]. All primers used in this study are listed in Table S1.

The MICs of CAZ-AVI were determined by using the broth microdilution method, and results were interpreted according to the clinical breakpoint (sensitive, ≤8/4 mg/L; resistant, ≥16/4 mg/L) recommended by Clinical Laboratory and Standards Institute (CLSI) [31]. The MICs of other antibiotics were also determined, including meropenem (MEM), ampicillin (AMP), ampicillin-avibactam (AMP-AVI), aztreonam (ATM), aztreonam-avibactam (ATM-AVI), polymyxin B (PB), gentamicin (GM), levofloxacin (LVX), ceftriaxone (CRO), CAZ, and tigecycline (TGC). All antibiotics were purchased from Meilunbio (Dalian, China). *K. pneumoniae* ATCC 700603 and *Escherichia coli* ATCC 25922 were used for quality control.

### In vitro selection of CAZ-AVI-resistant isolates

Multipassage resistance selection was performed at low CAZ-AVI concentrations (1/2 × the MICs of original strains) as previously described with slight modifications [32]. For each strain, 4 μl of overnight culture was inoculated into 4 ml of LB broth supplemented with CAZ-AVI at half of the MIC for the strain. After incubation at 37°C with shaking (220 rpm) for 16 h (Passage 1), 4 μl of the resulting culture was used to inoculate 4 ml of fresh LB broth containing CAZ-AVI at half of the MIC for original strains and incubated at 37°C with shaking (220 rpm) for 16 h (Passage 2). This was repeated daily for a total of 50 passages. MIC changes in the induced strains were monitored using broth microdilution method every two passages and the ones with more than a two-fold increase were stored at −80°C for further experiments. All induced strains after 50 passages were also stored at −80°C. *K. pneumoniae* ATCC 700603 was continuously passaged in the absence of antibiotics and served as a negative control.

CAZ-AVI MIC for the induced strains at Passage 50 was tested again before subsequent experiments to see if the cold storage affects the susceptibility. Based on their MIC values, resistant strains after induction were henceforth named following the format of “original strain name – induced resistance (IR)” (e.g. P77-IR). Susceptible strains were named following the format of “original strain name – induced susceptibility (IS)” (e.g. P152-IS).

### Stability of CAZ-AVI resistance and cross-resistance

To estimate the stability of CAZ-AVI resistance for the IR strains and two IS strains (P152-IS and C4-IS), overnight cultures of these strains were inoculated 1:1000 in 4 ml of fresh LB broth for 20 passages. The MIC of CAZ-AVI for the passaged strains was determined every two passages to record any changes.

The MICs of other antibiotics against the induced strains were also examined (based on CLSI) and compared with that of the original strains [31]. Cross-resistance among induced strains was confirmed when the MIC values of antibiotics other than CAZ-AVI increased four-fold or more.

### Whole-genome sequencing (WGS) and bioinformatic analysis

WGS was performed for the wild-type strains and derived IR and two IS strains (P152-IS and C4-IS). Briefly, single colonies from an overnight agar plate were cultured in 4 ml of LB broth at 37°C for 16 h, and genomic DNA was extracted using a Bacterial DNA Kit D3350 (Omega Bio-Tek, USA). WGS was conducted by Novogene (Beijing, China) using an Illumina Novaseq 6000 platform (Illumina, San Diego, CA, USA). The raw data were trimmed and assembled by shovill [33]. Prokka was used to annotate the assembled contigs [34]. Resistance genes were identified by ABRicate using NCBI database [35]. Multi-locus sequence typing (MLST) was performed by using MLST software [30]. Capsular serotypes (K) were identified by *wzi* typing using Bacterial Isolate Genome Sequence Database (BIGSdb) [36,37]. Single nucleotide polymorphisms (SNPs) were identified using Snippy [38]. To identify the passages in which the mutations occurred in the mutant strains, PCR were used to amplify target genes and PCR products were Sanger-sequenced by BGI (Beijing, China).

### Complementation experiment

Selected genes with mutations that occurred in multiple strains or had been previously reported were cloned into vector pACYC184 as described preciously [39,40] and transformed into appropriate strains to verify the role of mutations. For the validation of *bla*_KPC_, variants were amplified by PCR using primers (Table S1) designed according to the User Manual of In-Fusion® HD Cloning Kit (Takara, Tokyo, Japan). PCR products were purified using a gel extraction kit (Takara, Tokyo, Japan) and then cloned into the linearized plasmid pACYC184 digested by *Eco*RI or *Bam*HI using the In-Fusion® HD Cloning Kit. The resulting constructs were transformed by electroporation into the wild-type strains, the nucleotide sequences of the inserts were verified by Sanger sequencing (BGI, Beijing, China). For the validation of mutant *lamB* and *ftsI*, plasmids carrying the wild-type *lamB* or *ftsI* were constructed as described above and then transformed into mutant strains by electroporation. As a control, the empty vector was also introduced into the wild-type stains or mutant strains. The MIC assays for the complementary strains were performed in triplicate by broth microdilution with Mueller-Hinton (MH) broth.

### Determination of transcription levels

For induced strains with at least a four-fold increase in the MIC values of CAZ-AVI, the transcription levels of *bla*_KPC_ of all passages were measured_._ For each strain, overnight culture was inoculated 1:1,000 into 4 ml of fresh LB broth with CAZ-AVI at 1/2 of the MIC for the original strain and cultured at 37°C with 220 rpm shaking until the growth reached the logarithmic growth phase. Total RNA was isolated using a Bacteria RNA Extraction Kit (Vazyme, Nanjing, China) and cDNA was produced using a HiScript III-RT SuperMix for qPCR kit (Vazyme, Nanjing, China) according to the manufacturer's instructions. RT–PCR was performed using a ChamQ Universal SYBR qPCR Master Mix (Vazyme, Nanjing, China) on a LightCycle® 96 (Roche) using *bla*_KPC_ primers (Table S1). The relative transcript levels were calculated using the 2^-ΔΔCT^ method [41] with *rpoB* as the reference (Table S1). The average transcript levels were calculated from at least three independent RNA samples isolated from three separate microbial broth cultures for each strain.

The previous study showed that LamB plays an important role in OmpK35/OmpK36-defective and OmpK36-defective strains, thus, the transcription levels of proins (*ompK35*, *ompK36*, and *ompK37*) were also detected to explore the role of LamB [42] using the method described above.

## Statistical analysis

Continuous data were reported as means ± standard deviation (SD) and analyzed using Student’s *t*-test with SPSS software package (version 17.0, Chicago, IL). *P* values < 0.05 were regarded as statistically significant.

## Accession numbers

All genome sequencing data for this work were deposited at NCBI under BioProject accession no. PRJNA740115 (https://www.ncbi.nlm.nih.gov/bioproject/PRJNA740115) with BioSample SAMN19820424.

## Results

### Characteristics of bacterial strains

Fourteen KPC-KP strains isolated in China were included in this study based on their STs and serotypes [43,44]. As shown in Table 1, four strains were isolated from sputum samples, four from blood samples, three from urine samples, and three from sterile body fluids. Of the 14 KPC-Kp strains, 13 carried *bla*_KPC-2_ and one carried *bla*_KPC-12_. MLST revealed that 10 strains belonged to ST11, two to ST15, one to ST37 and one to ST1296. Among the ST11 strains, six had a capsular serotype of K47 and four of K64. The serotypes of the ST15, ST37, and ST1296 strains were K19, K12, and K75, respectively. All β-lactamases identified in the 14 strains are listed in Table S2.

**Table 1.** Characteristics of the strains used in this study.

Strain	Sample source	Carbapenemase types	Serotype	MLST
220	sputum	KPC-2	K47	11
1419	sputum	KPC-2	K47	11
14192	sputum	KPC-2	K47	11
BL94	sputum	KPC-2	K47	11
1295	blood	KPC-2	K64	11
BL18	blood	KPC-2	K64	11
BL152	blood	KPC-2	K64	11
84082	blood	KPC-2	K64	11
P77	urine	KPC-2	K47	11
C4	urine	KPC-12	K47	11
Q38	urine	KPC-2	K19	15
Q35	aseptic humoral	KPC-2	K19	15
P152	aseptic humoral	KPC-2	K75	1296
Q30	aseptic humoral	KPC-2	K12	37

Note: KPC, *Klebsiella pneumoniae* carbapenemase; MLST, multi-locus sequence typing.

As shown in Table 2, all of the 14 strains were sensitive to CAZ-AVI and TGC but demonstrated high MICs (≥64 mg/L) to ATM, AMP, CAZ, and CRO. Almost all strains were resistant to GM (85.7%) and LVX (92.8%) and most of strains were sensitive to PB (71.4%).

**Table 2.** Minimum inhibitory concentration (MIC) of various antibiotics for strains before and after induction.

Strain ID	MIC value (mg/L)^b^																							
	CAZ-AVI		MEM		AMP		AMP-AVI		ATM		ATM-AVI		PB		GM		LVX		CRO		TGC		CAZ	
	0^a^	50^a^	0^a^	50^a^	0^a^	50^a^	0^a^	50^a^	0^a^	50^a^	0^a^	50^a^	0^a^	50^a^	0^a^	50^a^	0^a^	50^a^	0^a^	50^a^	0^a^	50^a^	0^a^	50^a^
220	4	4	**≥64**	**≥64**	**≥128**	**≥128**	128	128	**>128**	**>128**	2	2	2	2	**≥128**	**≥128**	**16**	**16**	**≥128**	**≥128**	0.25	0.25	**1024**	**1024**
1419	4	**128**	**≥64**	2	**≥128**	**≥128**	128	256	**>128**	**>128**	4	32	**64**	**16**	**≥128**	**≥128**	**32**	**32**	**≥128**	**≥128**	0.25	0.25	**1024**	**2048**
14192	4	**32**	**≥64**	**≥64**	**≥128**	**≥128**	128	512	**>128**	**≥128**	4	16	**64**	**16**	**≥128**	**≥128**	**32**	**32**	**≥128**	**≥128**	0.25	0.25	**1024**	**>4096**
BL94	2	**16**	**≥64**	**32**	**≥128**	**≥128**	128	64	**>128**	**>128**	2	2	0.5	0.5	**≥128**	**≥128**	**≥128**	**32**	**≥128**	**≥128**	1	0.5	**1024**	**512**
1295	4	8	**≥64**	**≥64**	**≥128**	**≥128**	128	128	**>128**	**>128**	2	4	1	1	**≥128**	**≥128**	**64**	**64**	**≥128**	**≥128**	0.25	0.25	**512**	**512**
BL18	4	**32**	**≥64**	**≥64**	**≥128**	**≥128**	256	1024	**>128**	**>128**	4	8	0.5	0.25	**≥128**	**≥128**	**64**	**64**	**≥128**	**≥128**	1	0.5	**1024**	**4096**
BL152	4	**32**	**≥64**	**≥64**	**≥128**	**≥128**	128	512	**>128**	**>128**	4	32	2	**4**	**≥128**	**≥128**	**64**	**32**	**≥128**	**≥128**	1	2	**512**	**>4096**
84082	4	**32**	**≥64**	**≥64**	**≥128**	**≥128**	128	64	**>128**	**>128**	4	4	**32**	**32**	**≥128**	**≥128**	**32**	**16**	**≥128**	**≥128**	0.5	1	**1024**	**2048**
P77	4	**16**	**≥64**	**16**	**≥128**	**≥128**	128	64	**>128**	**>128**	2	2	1	1	**≥128**	**≥128**	**32**	**32**	**≥128**	**≥128**	0.5	0.25	**512**	**1024**
C4	2	8	**32**	**16**	**≥128**	**≥128**	64	32	**>128**	**>128**	2	2	0.5	0.5	**≥128**	**≥128**	**32**	**64**	**≥128**	**≥128**	0.25	0.25	**512**	**512**
Q38	1	**128**	**16**	**≥64**	**≥128**	**≥128**	128	64	**>128**	**>128**	≤0.125	2	0.5	0.25	**128**	**≥128**	**4**	**8**	**≥128**	**≥128**	0.25	0.25	**128**	**512**
Q35	0.5	**16**	**16**	**16**	**≥128**	**≥128**	128	64	**>128**	**>128**	1	1	1	0.5	**≥128**	**128**	**4**	**8**	**≥128**	**≥128**	0.25	0.5	**128**	**512**
P152	0.25	4	**16**	0.125	**≥128**	**≥128**	64	8	**>128**	**>128**	≤0.125	0.25	**16**	2	0.5	0.5	≤0.125	≤0.125	**≥128**	**>128**	0.5	0.25	**64**	**128**
Q30	2	**16**	**≥64**	**4**	**≥128**	**≥128**	128	8	**>128**	**128**	1	1	0.25	0.5	0.5	1	**32**	**64**	**≥128**	**≥128**	0.25	0.25	**128**	**512**

Note: Abbreviations: CAZ-AVI: Ceftazidime-avibactam; MEM: Meropenem; AMP: Ampicillin; AMP-AVI: Ampicillin-avibactam; ATM: Aztreonam; ATM-AVI: Aztreonam-avibactam; PB: Polymyxin B; GM: Gentamicin; LVX: Levofloxacin; CRO: Ceftriaxone; TGC: Tigecycline. ^a^ 0 indicates strains before *in vitro* induction; 50 indicates induced strains after 50 passages.^b^ MIC values above resistance breakpoint are shown in bold according to CLSI. The MIC values of AMP-AVI and ATM-AVI are not in bold because their resistance breakpoints are not available. The MIC results of tigecycline were determined according to the US Food and Drug Administration standard.

### In vitro selection of CAZ-AVI-resistant strains

After 50 passages, 10 of the 14 KPC-Kp strains became CAZ-AVI resistance with at least a 4-fold increase in the MIC value (Table 2). Strain P152 and C4 was considered susceptible according to the CLSI standard, despite a 16-fold and 4-fold increase in MIC respectively [31]. The MIC values increased eight-fold for most strains (50%), followed by 32-fold (16.7%) and four-fold (16.7%). The maximum increase in MIC of CAZ-AVI was 128-fold. Two strains (220 and 1295) had no significant change in MIC values after 50 passages and still susceptible to CAZ-AVI. As shown in Table 2, the MIC values for CAZ alone increased more than four folds in six strains, two folds in four other strains. In addition, three strain showed no change in MIC values of CAZ and one showed a 0.5-fold decrease.

As shown in Table 3, nine strains (64.3%) showed a significant increase in MICs of CAZ-AVI within 14 passages, and five of the 10 IR strains evolved into CAZ-AVI-resistant strains within 14 passages. During the induction process, the MIC of CAZ-AVI for most strains increased gradually, about two- to four-fold each passage (Table 3).

**Table 3.** Resistance to CAZ-AVI increased in a stepwise manner following passage with CAZ-AVI.

Strain ID	Minimum inhibitory concentration (MIC) of each passage (mg/L)																									
	0	2	4	6	8	10	12	14	16	18	20	22	24	26	28	30	32	34	36	38	40	42	44	46	48	50
ATCC 700603^a^	0.5	0.5	0.5	0.5	0.5	0.5	0.5	0.5	0.5	0.5	0.5	0.5	0.5	0.5	0.5	0.5	0.5	0.5	0.5	0.5	0.5	0.5	0.5	0.5	0.5	0.5
220	4	4	4	4	4	4	4	4	4	4	4	4	4	4	4	4	4	8	8	8	4	8	4	8	8	8
1419	4	16	16	8	8	16	16	16	32	32	32	32	32	64	32	32	32	32	32	64	32	32	32	32	64	128
14192	4	8	16	32	16	32	32	16	32	32	32	32	32	32	32	32	32	32	32	32	32	32	32	32	32	32
BL94	2	2	2	8	8	8	8	16	16	16	16	32	16	16	16	16	16	16	16	16	16	16	16	16	16	16
1295	4	8	4	4	4	4	4	4	4	8	4	4	4	8	8	4	4	8	4	4	8	8	8	8	8	8
BL18	4	4	8	8	8	8	8	16	16	16	16	16	16	16	16	16	16	32	16	32	16	16	16	16	32	32
BL152	4	4	8	8	8	16	8	8	8	8	16	8	16	8	16	16	16	16	32	32	32	32	32	32	32	32
84082	4	4	4	8	8	8	8	8	8	8	8	16	8	16	8	8	8	16	16	16	32	32	64	128	128	128
P77	2	2	2	4	4	2	4	4	2	2	2	2	2	2	2	2	2	4	2	2	2	4	4	8	16	16
C4	2	4	2	4	4	8	4	8	8	8	8	8	8	16	8	16	8	16	16	16	16	16	16	16	16	16
Q38	1	2	64	64	64	64	64	64	64	64	64	64	64	64	64	64	128	64	128	128	64	128	128	128	128	128
Q35	0.5	1	1	2	2	2	1	2	1	2	2	1	1	2	2	2	2	2	2	4	2	2	2	2	2	16
P152	0.25	0.5	0.5	0.5	0.5	0.5	1	2	2	2	4	1	4	2	1	2	2	2	4	4	4	4	4	4	4	4
Q30	2	2	1	2	1	1	1	2	1	2	2	1	2	2	2	2	2	2	2	2	2	2	2	2	2	16

^a^ ATCC 700603, the recommended strain by the Clinical & Laboratory Standards Institute for the MIC determination of CAZ-AVI, was passaged alongside the test strains in LB broth without antibiotics, acting as a negative control.

### Stability of induced resistance and occurrence of cross-resistance

To examine the stability of CAZ-AVI resistance developed after the *in vitro* selection, 10 IR strains and 2 IS strains (P152-IS and C4-IS) were subjected to further passages in the absence of antibiotics. The results showed that 9 strains maintained the resistance without any change in the MIC values after 20 passages. The MIC value for CAZ-AVI decreased by half in the other three strains, BL18-IR, BL152-IR, and Q38-IR, but remained resistant (Table S3).

The occurrence of cross-resistance was investigated by comparing the MIC values of antibiotics for the wild-type and induced strains. Two strains (BL152-IR, 14192-IR) showed cross-resistance to ATM-AVI and AMP-AVI with at least a four-fold increase in the MIC values. In addition, three strains (1419-IR, P152-IS, and Q38-IR) showed cross-resistance to ATM-AVI only (Table 2). Interestingly, the MIC of MEM for three strains (BL94-IR, Q30-IR, and P77-IR) decreased by four-fold or more though remained resistant, and two strains (P152-IS and 1419-IR) became susceptible to MEM (Table 2).

### Mutations occurred during induction

All mutations found in the ten IR strains and strains P152-IS and C4-IS are listed in Tables S4 and S5. Notably, 5 of these 12 induced strains had various mutations in porin LamB, 6 strains had diverse mutations in KPC-2 or KPC-12 and one strain had a mutated PBP3. Three unreported mutations had also been identified, an A172V in KPC-2 in strain P77-IR, a R178S in KPC-12 in strain C4-IS and an L367Q in PBP3 in strain 84082-IR. The mutations found in KPCs, PBP3, and porin LamB are listed in Table 4.

**Table 4.** Mutations identified in KPCs, LamB and PBP3 and expression level of *bla*_KPC_ during induction.

Strains	Mutations^a^				Relative expression of *bla*_KPC_ (P50/P0)^b^(means ± SD)	Change of MICs^c^ of CAZ-AVI (mg/L)
	KPC ^d^		porin LamB	PBP3		
	KPC-2	KPC-12				
BL94	E165_L166ins	-	R374L	W	0.86 ± 0.28	2→16
C4	-	R178S	R33H	W	1.33 ± 0.63	2→8
1419	L169P + S181 ins	-	W	W	1.12 ± 0.38	4→128
P77	A172V	-	R374L	W	1.26 ± 0.15	4→16
P152	E166_L167del	-	W	W	1.96 ± 1.19	0.25→4
Q30	E166_L167del	-	W	W	1.38 ± 0.40	2→16
BL152	W	-	W	W	2.45 ± 0.22	4→32
Q35	W	-	R374S	W	3.27 ± 0.04	0.5→16
Q38	W	-	R134P	W	2.05 ± 0.11	1→128
84082	W	-	W	L367Q	0.79 ± 0.21	4→32
BL18	W	-	W	W	5.83 ± 1.48	4→32
14192	W	-	W	W	1.40 ± 0.17	4→32

^a^ W,wild-type.

^b^ P50: strain after 50 passages; P0: strain before passages; the relative expression of *bla*_KPC_ was calculate by considering the *bla*_KPC_ expression at P0 as 1 for each strain; qRT-PCR data (relative expression of *bla*_KPC_) are given as means ± standard deviation (SD) of the results from three independent experiments.

^c^ Change in MIC in each strain is presented as ‘MIC of P0 → MIC of P50’.

^d^ -, indicates gene not present in strain.

The number of passages needed for the induction of mutations in KPCs, LamB and PBP3 were determined by identifying changes in these genes by PCR and Sanger sequencing. As shown in Table S6, in most passages, a new mutation would lead to a slightly higher MIC value. The MIC of passage in which the KPC-2 mutation occurred in Q30 increased the most (an eight-fold increase), and the MIC values of passages of other strains in which the mutations of KPC, PBP3 and LamB occurred were slightly increased (two- to four-fold).

### Roles of mutations in KPCs, PBP3, and LamB were confirmed by complementation

To explore the contribution of identified mutations to CAZ-AVI resistance, the wild-type *lamB* and *ftsI* genes were cloned into pACYC184, and the resulting constructs were transformed by electroporation into CAZ-AVI-resistant strains. Vectors carrying the mutant *bla*_KPC_ were electroporated into the wild-type CAZ-AVI-sensitive strains (Table S7).

Wild-type *lamB* was successfully transformed into 4 out of the 5 induced strains with a mutation in *lamB*. One of the strains, Q35-IR, was excluded from the experiment because its multidrug resistant nature that limited the availability of antibiotics for transformant screening. The MIC values of CAZ-AVI for three of the transformants derived from strains BL94-IR, C4-IS, and Q38-IR decreased two-fold, indicating that lamB mutations partially contributed to the induced CAZ-AVI resistance (Table 5). These above three strains also showed decreased MIC values of CAZ as well (0.5-fold). The recombinant plasmid carrying the wild-type *ftsI* gene reduced the MIC of the CAZ-AVI from 128 mg/L to 4 mg/L and the MIC of CAZ from 2048 mg/L to 1024 mg/L in the transformant derived from strain 84082-IR (Table 5).

**Table 5.** Complementation experiment for LamB and PBP3.

Transformed plasmid	Strain	MIC value of CAZ-AVI (mg/L)				MIC value of CAZ (mg/L)			
		Mutant strain	Mutant stain + empty vector	Mutant stain complemented with wild-type gene	MIC Fold change	Mutant strain	Mutant stain + empty vector	Mutant stain complemented with wild-type gene	MIC Fold change
pAC*lamB*	P77-IR	16	16	16	1	1024	1024	1024	1
	C4-IS	16	16	8	0.5	512	512	256	0.5
	BL94-IR	16	16	8	0.5	512	512	256	0.5
	Q38-IR	64	64	32	0.5	512	512	256	0.5
pAC *ftsI*	84082-IR	128	128	4	0.03	2048	2048	1024	0.5

Note: The CAZ-AVI resistant strains after induction were named following the format of ‘original strain name -IR’ (e.g. P77-IR). The induced strains that were still susceptible to CZA-AVI are named as ‘original strain name -IS’ (e.g. P152-IS).

As shown in Table 6, cloning of the six *bla*_KPC_ mutants into the wild-type strains increased their CAZ-AVI resistance by at least four-fold as demonstrated by the MIC values ranging from 8 mg/L to128 mg/L. Interestingly, these mutations in *bla*_KPC_ showed no obvious effect on the MIC of CAZ.

**Table 6.** Complementation experiment of KPCs.

Transformed plasmid	Strain	MIC value of CAZ-AVI (mg/L)				MIC value of CAZ (mg/L)			
		WT strain	WT strain + empty vector	WT strain complemented with gene of interest	MIC Fold change	WT strain	WT strain + empty vector	WT strain complemented with gene of interest	MIC Fold change
pACBL94KPC	BL94	2	2	16	8	1024	1024	1024	1
pACC4KPC	C4	2	2	8	4	512	512	512	1
pAC1419KPC	1419	4	4	128	32	1024	1024	1024	1
pACP77KPC	P77	2	2	8	4	512	512	1024	2
pACP152KPC	P152	0.25	0.25	32	128	64	64	128	2
pACQ30KPC	Q30	2	2	32	16	128	128	256	2

Note: The recombinant plasmid's target fragments containing *bla*_KPC_ were obtained from each CAZ-AVI resistant strain. WT strains, wild type strains.

### Gene expression analysis

**Figure 1.**
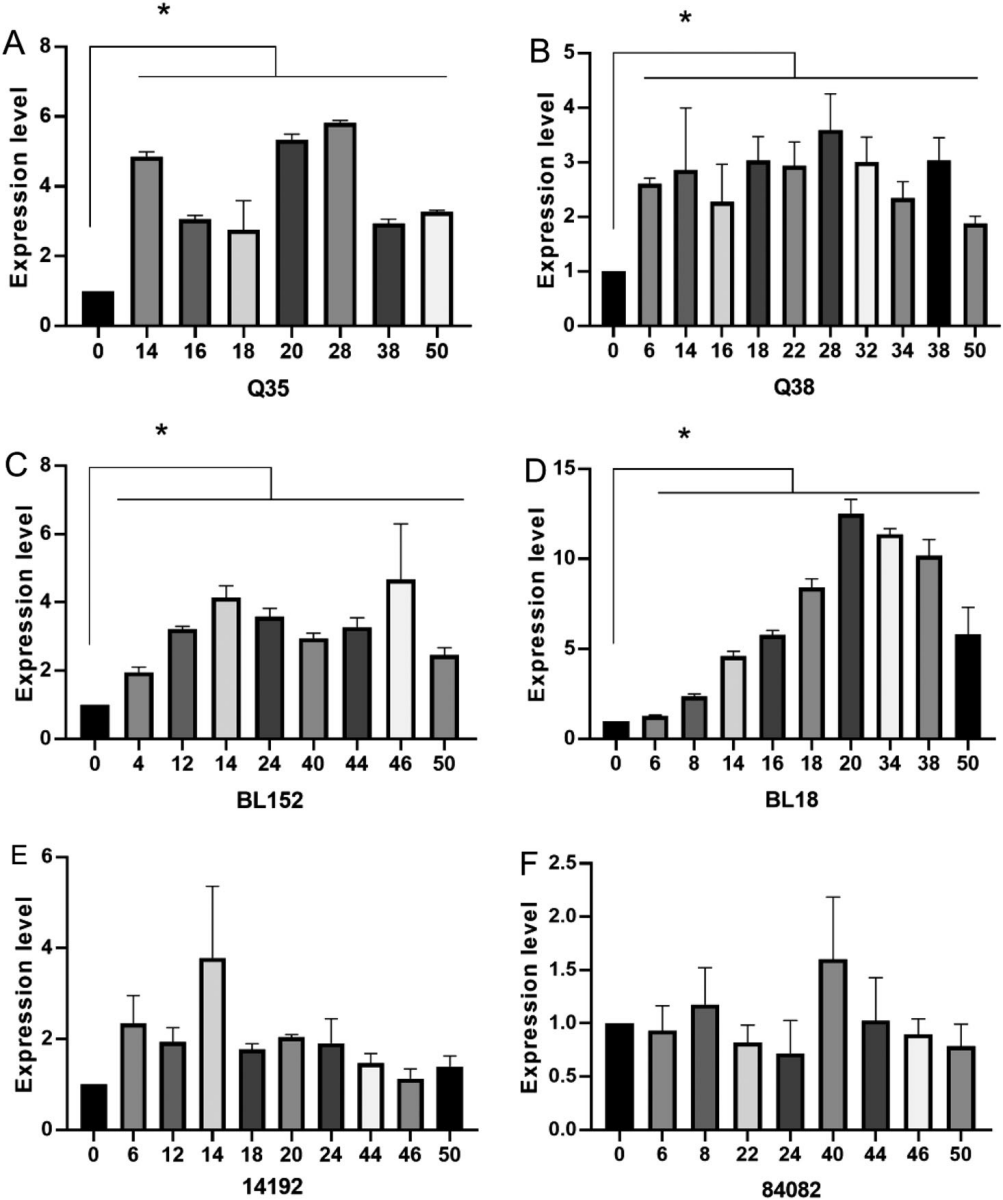
The *bla*_KPC_ expression levels in strains without mutations in KPC. The horizontal axis is the number of passages of induction. The vertical axis is the relative expression level compared to the strain before induction (expression = 1.0). The housekeeping gene *rpoB* was used as the endogenous reference gene. All RT-qPCR were carried out in triplicate. *P < 0.05 (Student’s *t*-tests).

Among strains confirmed with an at least four-fold increase in CAZ-AVI MIC, the *bla*_KPC_ expression in four strains (Q35-IR, Q38-IR, BL152-IR, and BL18-IR) was significantly increased (*P *< 0.05) compared with the pre-induction strains. Strains with an increased expression level of *bla*_KPC_ all showed significantly increased MIC values of CAZ, which increased by four-fold. The elevated transcription levels all appeared within fourteen passages and remained stable (Figure 1, Table 4).

Among the 10 IR strains and two IS strains (P152-IS and C4-IS), a significant decrease (*P *< 0.05) was found for the expression level of *ompK35*, *ompK36*, and *ompK37* in 4, 7 and 3 strains, respectively. Among the 5 strains with mutations in LamB, 4 strains (except P77) had a decrease in the expression level of *ompK35* or *ompK36* (Fig S1).

## Discussion

In this study, the resistance mechanism of KPC-Kp against CAZ-AVI was investigated by using *in vitro* induction of resistance at low antibiotic concentrations. Ten of the 14 strains became CAZ-AVI resistant after the *in vitro* selection. Apart from developing resistance to CAZ-AVI after induction, some strains also showed cross-resistance to ATM-AVI, AMP-AVI and CAZ. It was reported that mutations in KPC may decrease the inhibition effect of avibactam [27] and changes in membrane permeability and efflux pump function may affect multiple antibiotics [14,45,46]. Importantly, these strains remained resistant to CAZ-AVI after a further 20 passages in the absence of antibiotics, demonstrating the stability of the induced resistance. Comparative genomic analysis revealed different mutations in porin LamB, KPCs and PBP3 in the IR (induced) strains (Table 4), suggesting these mutations may be responsible for the developed CAZ-AVI resistance.

Previous studies demonstrated that the distinctive outer membrane barrier with outer membrane proteins (OMPs) in gram-negative bacteria can lead to a high resistance to several antibiotics [45,46]. Yang et al. reported that the mutations in OmpK35/36, common porins in *K. pneumoniae*, invalidated AVI diffusion across the outer membrane, leading to a significant increase in the MIC of CAZ-AVI (4–32 mg/L) [14]. Maltoporin LamB, involved in the transportation of maltose and maltodextrins, is an 18-stranded β-barrels trimer located in the outer membrane of *E. coli* and *K. pneumoniae* [47–49]. Previous studies demonstrated that overexpression of LamB compensates the loss of the major OmpK36 and contributes to high levels of resistance to various classes of antibiotics in *K. pneumoniae* [42,49]. To our best knowledge, here we report for the first time that different mutations in porin LamB were involved in CAZ-AVI resistance in the KPC-Kp strains and may mainly affect the MICs of CAZ. In mutant strains, complementary expression of wild-type LamB reduced the MIC value of CAZ-AVI, and the expression levels of *ompK35* or *ompK36* were all significantly decreased in our study. The findings agree with previous reports that LamB has a more important role in OmpK35/OmpK36-defective and OmpK36-defective strains [42]. Three mechanisms may explain the change of CAZ-AVI resistance caused by LamB mutations according to previous studies: (1) *lamB* encodes a specific influx channel of antibiotics; (2) the lack of porin LamB influences the expression of some major porins in CRKP, such as OmpK35 and OmpK36; and (3) maltose may play an important role in antibiotic resistance by blocking the ability of bacteria to ingest maltose increases survival, and LamB affects the presence of maltose [49,50]. However, further studies are needed to confirm the underlying mechanism.

Interestingly, although expression of the wild-type LamB in the mutant strains reduced the MIC values of CAZ-AVI and CAZ, the changes were not significant (Table 5). This may be explained by that antibiotic resistance caused by porin mutations is usually synergistic with other mechanisms. Nelson et al. have reported that OmpK35/36 deficiency, combined with a significant increase in the expression level of *bla*_KPC_ and increased efflux activity resulted in an eight-fold increase in MIC in the KPC-Kp strain [14]. To evaluate whether mutation (T333N) in OmpK36 contributes to ceftazidime-avibactam resistance, Nelson et al. have transformed the wild-type OmpK36 into the mutant strain, and the MIC value was subsequently reduced by two-fold, which is similar to our results in this study [14]. Considering that only porin LamB was complemented in mutant strains, it is expected that the change in MIC values would not be significant. Alternatively, LamB is associated with a variety of antibiotics and has a non-specific effect on CAZ-AVI [42]. García-Sureda et al. reported that LamB knockout strains increased the MICs of antibiotics by about two-fold which is also in consistent with the results from our study.

Two KPC mutations in the Ω loop prompting resistance to CAZ-AVI in our study are previously unknown mutations [51]. These mutations in our study resulted in increased MICs to varying degrees [23,24]. We believe that mutations in KPC are diverse and frequent, posing a great threat to CAZ-AVI's effectiveness [23,24,28]. In addition, our complementation experiment results showed that different mutations in KPC may have no significant effect on the MIC values of CAZ (Table 6), indicating that mutations in KPC may mainly affected the activity of AVI. We also identified increased *bla*_KPC_ expression level in most strains without KPC mutations, revealing a probable mechanism leading to CAZ-AVI resistance in these strains. The increase of MIC caused by the enhanced expression level of *bla*_KPC_ was not as significant as that caused by KPC mutations. Notably, our results showed that the sensitivity to meropenem in strains with a mutant KPC was restored as previously reported [52,53] while that of strains with an increased expression level of *bla*_KPC_ was not, posing further obstacles for selection of appropriate clinical treatments. Studies investigating the mechanism of increased *bla*_KPC_ expression in CAZ-AVI resistance strains are currently limited. Two studies identified that an increase in *bla*_KPC_ copy number is responsible for the elevated *bla*_KPC_ expression level [14,54]. However, we had not identified the change of copy number of *bla*_KPC_ in our resistant strains and the specific mechanism explaining the elevated expression level in our study remains to be elucidated.

Additionally, a novel amino acid replacement (L367Q) in PBP3 was found to have caused a significant increase in MIC of CAZ-AVI in strain 84082-IR. PBP3 is the primary target of CAZ. It was reported that a four-amino-acid insertion (T-I-P-Y) caused an increase in CAZ-AVI resistance because the mutation decreased the affinity between PBP3 and the β-lactam CAZ [29,55]. Our results showed that this novel mutation contributed to the significant increase in CAZ-AVI resistance, indicating the need for a greater focus on PBP3 mutations which are not considered a priority [26].

In conclusion, we have identified and investigated multiple mechanisms that may be responsible for the developed CAZ-AVI resistance in KPC-Kp strains. Mutations in porin LamB may co-occur with other resistance mechanisms, such as mutations in KPCs and increased expression of *bla*_KPC_. In contrast, no KPC mutations were detected in any of the strains with elevated *bla*_KPC_ expression, suggesting that the two mechanisms may not always coexist.

## Summary

This was the first time that mutations in porin LamB were found to be associated with CAZ-AVI resistance. We show that mutations in the KPCs and PBP3 are diverse and can cause increases in CAZ-AVI MICs to different degrees. In strains without KPC mutations, the increase of *bla*_KPC_ expression may be an important mechanism contributing to CAZ-AVI resistance.

## Supplementary Material

_______final.doc
