# Supplementary material for: Mutations in porin LamB contribute to ceftazidime-avibactam resistance in KPC-producing Klebsiella pneumoniae
Source: Emerg Microbes Infect. 2021 Nov 2;10(1):2042–51. doi: 10.1080/22221751.2021.1984182 (PMC8567916; doi:10.1080/22221751.2021.1984182)
Supplement: _______final.doc [file TEMI_A_1984182_SM2985.doc]

Table S1: Primers used in this study.

| Experiment | Gene | Primer | Sequence (5'>3') |
| --- | --- | --- | --- |
| Complementation  expreiment | *KPC-EcorI* | KPC-eF | GCTCATCCGGAATTCTACGCAACTACAGTGACATAG |
|  |  | KPC-eR | TTTCATTGCCATACGAATGTCGAGGCGGTGGCAGTA |
|  | *KPC-BamHI* | KPC-bF | CCGTCCTGTGGATCCACCACAGCATTCCGCAAC |
|  |  | KPC-bR | CGGCGTAGAGGATCCCCTAAATGTGACAGTGGT |
|  | *lamB-EcorI* | LamB-eF | TGCCATACGGAATTCATAGCATCGGTTTAGCGG |
|  |  | LamB-eR | GCTCATCCGGAATTCGAAGGCGAGGTCCAGGTC |
|  | *lamB-BamHI* | LamB-bF | CCGTCCTGTGGATCCGGCTACCGGTGGACAGCG |
|  |  | LamB-bR | CGGCGTAGAGGATCCATAGCATCGGTTTAGCGG |
| qRT-PCR | *KPC-qpcr* | KPC-qF | GAACCTGCGGAGTGTATG |
|  |  | KPC-qR | TGTGCTTGTCATCCTTGTT |
|  | *rpoB-qpcr* | rpoB-qF | AACGAATATGGCTTCCTTGAGACGC |
|  |  | rpoB-qR | GTTCGCCTGAGCGATAACGTAGTTG |
|  | *AcrA-qpcr* | qAcrA-F | CTCTGGCGGTCGTTCTGAT |
|  |  | qAcrA-R | CCTGGCAGTTCGGTGGTTA |
|  | *TolC-qpcr* | qTolC-F | CGCCACCACTACGCTGTAT |
|  |  | qTolC-R | CCTAACGCCGACTTAATGTTCA |
|  | *PBP3-qpcr* | qPBP3-F | ATGCCGTCCTCAGCGTTAG |
|  |  | qPBP3-R | GGATATAAGCCACTGCGTTCTC |
|  | *PBP2-qpcr* | qPBP2-F | CCGTGGCGATGATTCTGGA |
|  |  | qPBP2-R | CTTGGCAGCGTAGTGTTGTTAT |
|  | *OmpK37-qpcr* | qompK37-F | GGCGACTCCTACACCTATGC |
|  |  | qompK37-R | CCTCCACCAGACCGAAGAA |
|  | *OmpK35-qpcr* | q-ompk35F | GCAGCGACGATACCACCTAT |
|  |  | q-ompk35R | GTCTGGGAACCTTCAACATTGG |
|  | *OmpK36-qpcr* | q-ompk36F | TGGACTCGTCTGGCATTCG |
|  |  | q-ompk36R | CAGGAAGTTGTCAGAACCGTAG |
|  | *AcrB-qpcr* | qAcrB-REF-F | CAATACGGAAGAGTTTGGCA |
|  |  | qAcrB-REF-R | CAGACGAACCTGGGAACC |
| Identification of SNPS | *KPC-snp* | KPC-snpF | ATGTCACTGTATCGCCGT |
|  |  | KPC-snpR | GGTGGTGGGCCAATAGAT |
|  | *lamB-snp* | lamB-snpF | CGCTTGTCGTCGGCTGCATA |
|  |  | lamB-snpR | AGGTAACTTCGCTGTCGTCACC |
|  | *ftsI-snp* | ftsI-snpF | GCTCAATACCGTGCCTTATCG |
|  |  | ftsI-snpR | GCCAATCGTTGCGTAGACTC |

Table S2 The β-lactamases in the strains

| Strains | β-lactamases | Strains | β-lactamases | Strains | β-lactamases |
| --- | --- | --- | --- | --- | --- |
| 1419 | SHV-187  SHV-12  SHV-158  SHV-187  KPC-2  TEM-1  SHV-187  CTX-M-65 | BL152 | SHV-187  SHV-12  SHV-158  SHV-187  SHV-187  LAP-2  KPC-2  TEM-1  CTX-M-65 | Q38 | KPC-2  SHV-106  TEM-1  CTX-M-15 |
| 14192 | SHV-187  SHV-12  SHV-158  SHV-187  KPC-2  TEM-1  SHV-187  CTX-M-65 | Q35 | SHV-106  CTX-M-15  TEM-1  KPC-2 | 84082 | SHV-187  SHV-12  SHV-158  SHV-187  SHV-187  LAP-2  KPC-2  TEM-1  CTX-M-65 |
| BL94 | SHV-11  SHV-158  SHV-12  SHV-187  SHV-187  KPC-2  TEM-1  CTX-M-65 | P77 | CTX-M-65  SHV-187  SHV-12  SHV-158  CTX-M-102  TEM-1  SHV-187  SHV-187  LAP-1  KPC-2  TEM-1 | P152 | SHV-187  KPC-2 |
| BL18 | SHV-187  SHV-12  SHV-158  SHV-187  SHV-187  LAP-2  KPC-2  TEM-1  CTX-M-65 | C4 | SHV-187  SHV-12  SHV-158  SHV-187  SHV-187  KPC-12  TEM-1 | Q30 | TEM-1  TEM-1  SHV-110  KPC-2  CTX-M-3  TEM-1 |

Table S3: MICs (mg/L) changes in induced strains during 20 passages in the absence of CAZ-AVI

| Strains | Passages | | | | | | | | | | |
| --- | --- | --- | --- | --- | --- | --- | --- | --- | --- | --- | --- |
|  | 0 | 2 | 4 | 6 | 8 | 10 | 12 | 14 | 16 | 18 | 20 |
| ATCC700603 | 0.5 | 0.5 | 0.5 | 0.5 | 0.5 | 0.5 | 0.5 | 0.5 | 0.5 | 0.5 | 0.5 |
| 1419 | 128 | 128 | 128 | 128 | 128 | 128 | 128 | 128 | 128 | 128 | 128 |
| 14192 | 32 | 32 | 32 | 32 | 32 | 32 | 32 | 32 | 32 | 32 | 32 |
| BL94 | 16 | 16 | 16 | 16 | 16 | 16 | 16 | 16 | 16 | 16 | 16 |
| BL18 | 32 | 32 | 32 | 32 | 16 | 16 | 16 | 16 | 16 | 16 | 16 |
| BL152 | 32 | 16 | 16 | 16 | 16 | 16 | 16 | 16 | 16 | 16 | 16 |
| 84082 | 32 | 32 | 32 | 32 | 16 | 16 | 16 | 16 | 16 | 16 | 16 |
| P77 | 16 | 16 | 16 | 16 | 16 | 16 | 16 | 16 | 16 | 16 | 16 |
| C4 | 16 | 16 | 16 | 16 | 16 | 8 | 8 | 8 | 8 | 8 | 8 |
| Q38 | 128 | 128 | 64 | 64 | 64 | 64 | 64 | 64 | 64 | 64 | 64 |
| Q35 | 16 | 16 | 16 | 16 | 16 | 16 | 16 | 16 | 16 | 16 | 16 |
| P152 | 4 | 4 | 4 | 4 | 4 | 4 | 4 | 4 | 4 | 4 | 4 |
| Q30 | 8 | 8 | 8 | 8 | 8 | 8 | 8 | 8 | 8 | 8 | 8 |

Table S4 Mutations identified in induced strains

| strains | mutations of proteins | proteins | Change of MICs(mg/ml) | Mutations of genes |
| --- | --- | --- | --- | --- |
| 1419 | R128H | Arginine transport ATP-binding protein ArtP | 4→128 | 383G>A |
|  | A291T | Endolytic peptidoglycan transglycosylase RlpA |  | 871G>A |
|  | N137S | Putative HTH-type transcriptional regulator LgoR |  | 410A>G |
|  | N97S | Aromatic amino acid transport protein AroP |  | 290A>G |
|  | N558S | Outer membrane protein assembly factor BamA |  | 1673A>G |
|  | V202A | Inner membrane ABC transporter permease protein YdcV |  | 605T>C |
|  | S245G | Major myo-inositol transporter IolT |  | 733A>G |
|  | S181ins | Carbapenem-hydrolyzing beta-lactamase KPC |  | 540_542dupATC |
|  | L169P | Carbapenem-hydrolyzing beta-lactamase KPC |  | 503T>C |
| 14192 | A78V | Plasmid-derived single-stranded DNA-binding protein ssb_3 | 4→32 | 233C>T |
|  | I5V | Glucose-1-phosphate thymidylyltransferase 2 rffH_2 |  | 13A>G |
| 84082 | L367Q | Peptidoglycan D,D-transpeptidase FtsI | 4→32 | 1100T>A |
|  | D567E | Putative tyrosine-protein kinase in cps region |  | 1701T>A |
| BL18 | N142fs | Endolytic peptidoglycan transglycosylase RlpA | 4→32 | 423_433delCAACGATCGGG |
|  | L248I | Diguanylate cyclase DgcP |  | 742C>A |
| BL94 | R374L | Maltoporin LamB | 2→16 | 1121G>T |
|  | E165_L166ins | Carbapenem-hydrolyzing beta-lactamase KPC |  | 499_504dupGAGCTG |
|  | V162A | L-threonate dehydrogenase ltnD |  | 485T>C |
| BL152 | P647L | Putative tyrosine-protein kinase in cps region | 4→32 | 1940C>T |
|  | M145L | IS5 family transposase IS903 |  | 433A>C |
|  | G220V | IS5 family transposase IS903 |  | 659G>T |
| C4 | R33H | Maltoporin LamB | 2→8 | 98G>A |
|  | T384I | Periplasmic alpha-amylase malS |  | 1151C>T |
|  | D327E | IS110 family transposase ISEc21 |  | 981C>A |
|  | R178S | Carbapenem-hydrolyzing beta-lactamase KPC |  | 529C>A |
| P77 | G202V | Sensor histidine kinase RcsC | 4→16 | 605G>T |
|  | R374L | Maltoporin LamB |  | 1121G>T |
|  | P69S | Protein PsiB |  | 205C>T |
|  | A172V | Carbapenem-hydrolyzing beta-lactamase KPC |  | 512C>T |
| P152 | L69fs | Inner membrane protein YabI | 0.25→4 | 206delT |
|  | R15H | Transcriptional regulatory protein OmpR |  | 44G>A |
|  | E166_L167del | Carbapenem-hydrolyzing beta-lactamase KPC |  | 499_504delGAGCTG |
|  | V45A | IS3 family transposase ISKpn1 |  | 134T>C |
| Q30 | I184S | Lactose operon repressor lacI_1 | 2→16 | 551T>G |
|  | F403S | Periplasmic alpha-amylase malS |  | 1208T>C |
|  | S274fs | Protein mlc |  | 820_821dupAG |
|  | E166_L167del | Carbapenem-hydrolyzing beta-lactamase KPC |  | 499_504delGAGCTG |
| Q35 | G119C | Cyclic di-GMP-binding protein bcsB_2 | 0.5→16 | 355G>T |
|  | R374S | Maltoporin LamB |  | 1120C>A |
| Q38 | D295V | Maltose/maltodextrin import ATP-binding protein MalK | 1→128 | 884A>T |
|  | R134P | Maltoporin LamB |  | 401G>C |

Table S5 Mutation of proteins related to β-lactam resistance

| strains | Mutations^a^ | | | | | | | |
| --- | --- | --- | --- | --- | --- | --- | --- | --- |
|  | OmpK35 | OmpK36 | OmpK37 | PBP2 | PBP3 | AcrA | AcrB | TolC |
| 1419 | W | W | W | W | W | W | W | W |
| 14192 | W | W | W | W | W | W | W | W |
| BL94 | W | W | W | W | W | W | W | W |
| BL18 | W | W | W | W | W | W | W | W |
| BL152 | W | W | W | W | W | W | W | W |
| 84082 | W | W | W | W | L367Q | W | W | W |
| P77 | W | W | W | W | W | W | W | W |
| C4 | W | W | W | W | W | W | W | W |
| Q38 | W | W | W | W | W | W | W | W |
| Q35 | W | W | W | W | W | W | W | W |
| P152 | W | W | W | W | W | W | W | W |
| Q30 | W | W | W | W | W | W | W | W |

^a^ Mutations are obtained by comparing the induced strains (passage 50) with the original strains (Passage 0). “W” means wild type.

Table S6 Mutant passages of each strain and protein

| Strains | Passages of mutations^ab^ | | | | | | | | | | | |
| --- | --- | --- | --- | --- | --- | --- | --- | --- | --- | --- | --- | --- |
|  | KPC | | | | | | Porin LamB | | | PBP3 | | |
|  | KPC-2 | | | KPC-12 | | | mutation | passages | Increase fold of MICs^c^ | mutation | passages | Increase fold of MICs^c^ |
|  | mutation | passages | Increase fold of MICs^c^ | mutation | passages | Increase fold of MICs^c^ |  |  |  |  |  |  |
| BL94 | E165_L166ins | 6 | 4 | - | - | - | R374L | 14 | 2 | W | - | - |
| C4 | - | - | - | R178S | 26 | 1-2 | R33H | 34 | 1-2 | W | - | - |
| 1419 | L169P | 10 | 2 | - | - | - | W | - | - | W | - | - |
| 1419 | S181 ins | 50 | 2 | - | - | - | W | - | - | W | - | - |
| P77 | A172V | 46 | 2 | - | - | - | R374L | 48 | 2 | W | - | - |
| P152 | E166_L167del | 36 | 2 | - | - | - | W | - | - | W | - | - |
| Q30 | E166_L167del | 50 | 8 | - | - | - | W | - | - | W | - | - |
| Q35 | W | - | - | - | - | - | R374S | 26 | 2 | W | - | - |
| Q38 | W | - | - | - | - | - | R134P | 32 | 1-2 | W | - | - |
| 84082 | W | - | - | - | - | - | W | - | - | L367Q | 46 | 2 |

^a^ W: wild-type

^b^-, does not exist.

^c^ Changes in MIC are acquired by comparing MICs of strains in 0 passages and 50 passages.


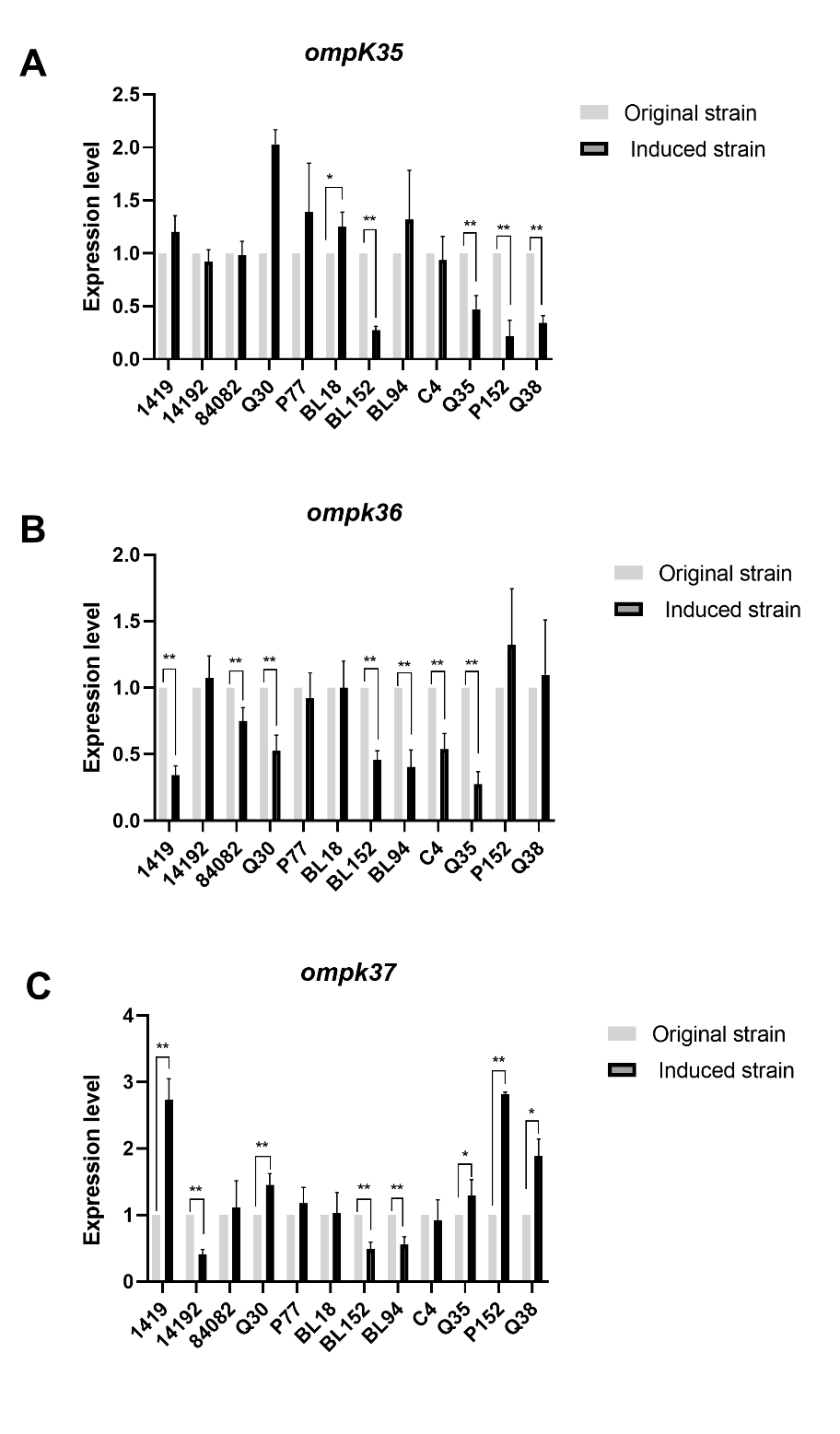


Figure S1. The expression levels of strains without mutations in *ompK35*, *ompK36* and *ompK37*. The housekeeping gene *rpoB* was used as the endogenous reference gene. The original strain was used as the reference strain (expression = 1.0). All RT-qPCR were carried out in triplicate. **P*< 0.05; ** *P* < 0.01 (Student’s *t*-tests).

Table S7: Complementation experiment.

| Genes | Strain that provided target fragment | Strains transformed |
| --- | --- | --- |
| *bla*_KPC_ | BL94-IR | BL94 |
|  | C4-IS | C4 |
|  | 1419-IR | 1419 |
|  | P77-IR | P77 |
|  | P152-IS | P152 |
|  | Q30-IR | Q30 |
| *lamB* | P77 | P77-IR |
|  | C4 | C4-IS |
|  | BL94 | BL94-IR |
|  | Q38 | Q38-IR |
| *ftsI* | 84082 | 84082-IR |

Note: Based on their MIC values, resistant strains after induction were henceforth named following the format of ‘original strain name - induced resistance (IR)’ (e.g. P77-IR). Susceptible strains were named following the format of ‘original strain name - induced susceptibility (IS)’ (e.g. C4-IS).
